# The Mediating Factor of Immune Cell in the Causal Relationship Between Cardiovascular Disease‐Related Plasma Proteins and Parkinson’s Disease: A Network Mendelian Randomization Analysis

**DOI:** 10.1155/mi/7919308

**Published:** 2026-03-04

**Authors:** Ruotong Yao, Siyao Chen, Yangguang Lu, Yiquan Li, Bohuai Yu, Yingying Liu, Tingxuan Zhang, Yusheng Zhu, Feng Chen, Yuhan Lin, Yukai Wang, Cai Li

**Affiliations:** ^1^ Department of Neurology, Taizhou Hospital of Zhejiang Province, Wenzhou Medical University, 150 Ximen Road Linhai City, Taizhou, Zhejiang, China, wmu.edu.cn; ^2^ The First School of Medicine, School of Information and Engineering, Wenzhou Medical University, Wenzhou, Zhejiang, China, wmu.edu.cn; ^3^ Renji College, Wenzhou Medical University, Wenzhou, Zhejiang, China, wmu.edu.cn; ^4^ The Second School of Medicine, Wenzhou Medical University, Wenzhou, Zhejiang, China, wmu.edu.cn

**Keywords:** cardiovascular disease, immune phenotype, Mendelian randomization, Parkinson’s disease

## Abstract

**Background:**

Cardiovascular diseases (CVDs) and their associated plasma proteins exhibit significant correlations with immunity and Parkinson’s disease (PD). However, the specific contributions to the risk of developing PD remain unclear. This study aims to investigate the potential causal relationship between CVD‐related plasma proteins and the risk of PD and explore the significant mediating role of immune cell phenotypes.

**Methods:**

Using publicly available genetic data, we conducted Mendelian randomization (MR) analysis using the inverse variance weighting method to explore the causal relationship between 83 CVD‐related plasma proteins and PD. Various MR analysis models were employed for sensitivity analysis. Concurrently, we utilized bioinformatics methods such as protein–protein interaction networks and pathway enrichment analysis to investigate the potential associations between CVD‐related plasma proteins and PD‐related genes. Finally, we employed a mediator MR design to identify the mediating effects of 731 immune cell phenotypes in the onset of PD.

**Results:**

Elevated levels of Fas cell surface death receptors (*p* = 0.015) and nerve growth factor (*p* = 0.026) are associated with a reduced risk of PD, while increased levels of thrombomodulin (*p* = 0.028) are a risk factor for PD. Four immune phenotypes play a significant mediating role in the association between CVD‐related proteins and the pathogenesis of PD. Sensitivity analysis indicates that the results are robust.

**Conclusions:**

Our study elucidates the close genetic association between CVD‐related plasma proteins and PD and identifies the significant mediating role of immune cells, thereby providing valuable insights for future research and clinical applications.

## 1. Introduction

Parkinson’s disease (PD) is the second most common neurodegenerative disorder, characterized by the loss of dopaminergic neurons in the substantia nigra and the accumulation of α‐synuclein, along with hallmark motor symptoms such as tremors, bradykinesia, and rigidity [1]. Over the past generation, the global burden of PD has more than doubled due to an increase in the elderly population [2], making it crucial to thoroughly explore the pathogenesis of PD and its contributing factors. Meanwhile, cardiovascular diseases (CVDs) predominantly manifest as age‐associated conditions. A systematic analysis highlighted CVDs as prominent contributors to the burden of noncommunicable diseases [3].

Li et al. [4] reported the prevalence of stroke and coronary artery disease in two distinct population cohorts, revealing that these conditions may be potential components of the PD pathogenesis. Furthermore, a recent study further demonstrated a causal relationship between PD and increased risk of CVDs and stroke, indicating a significant association between these diseases [5]. Such findings intimate that PD may not only coexist with cardiovascular issues but also actively promote their development. The global disease burden of PD and CVDs cannot be underestimated, stimulating a burgeoning interest in the interplay between these two diseases, hinting at a potential correlation.

Folkersen et al. [6] proposed 83 plasma proteins associated with CVDs in 2017, suggesting their potential as biomarkers for predicting cardiovascular risk. Regarding the relationship between PD and plasma proteins, a recent study analyzed data from the UK Biobank and identified a set of 22 plasma proteins that significantly improved the prediction of PD onset, indicating their potential utility in early identification of high‐risk individuals [7]. Plasma proteins as biomarkers hold significant promise, potentially enhancing early diagnosis and informing treatment strategies. Combining proteomics data with clinical assessments can significantly enhance the identification of high‐risk individuals for PD and CVDs, leading to a better understanding of the underlying mechanisms of the diseases.

Previous studies have elucidated the potential correlation of the immune‐inflammation axis in neurological disorders such as epilepsy [8], multiple sclerosis [9], and Alzheimer’s disease (AD) [10]. In the context of PD, PD is characterized by a low level of systemic inflammation, which may be due to the aberrant activation of the immune system [11]. Neuroinflammation emerges as a pivotal player in the pathophysiology of PD, and many immune cells, including but not limited to microglia, T cells, B cells, and NK cells, contribute to neuroinflammation by releasing inflammatory mediators and interacting with each other. This cascade ultimately culminates in the demise of neuronal cells, directly or indirectly, significantly influencing the progression of PD [12, 13]. The cardiovascular system contains a variety of immune cells, including macrophages, T cells, and B cells, which contribute to homeostasis and pathology [14]. Past research has definitively established the profound causal link between these immune cells and the onset as well as the prognosis of CVDs, underscoring the pivotal importance of balancing these immune responses [15, 16].

Based on the aforementioned evidence, we hypothesize that there is a significant causal relationship between CVD‐related proteins and PD, which is mediated by specific immune cell phenotypes. Given the undeniable role of immune cells in both realms, delving deeper into their potential mediating role between the two becomes imperative. In this study, we comprehensively explore the potential causal relationship between cardiovascular and cerebrovascular disease‐related proteins and the risk of PD and investigate the significant mediating role of immune cell phenotypes.

## 2. Materials and Methods

### 2.1. Study Design

This study is designed using Mendelian randomization (MR). MR is a research method that uses genetic variations as natural experiments to provide evidence regarding the presumed causal relationships between modifiable risk factors and diseases [17]. This method minimizes the potential bias from confounding and reverse causality in epidemiological studies [18]. This study adhered to the Strengthening the Reporting of Observational Studies in Epidemiology Using MR (STROBE‐MR) guidelines [18].

**Figure 1:**
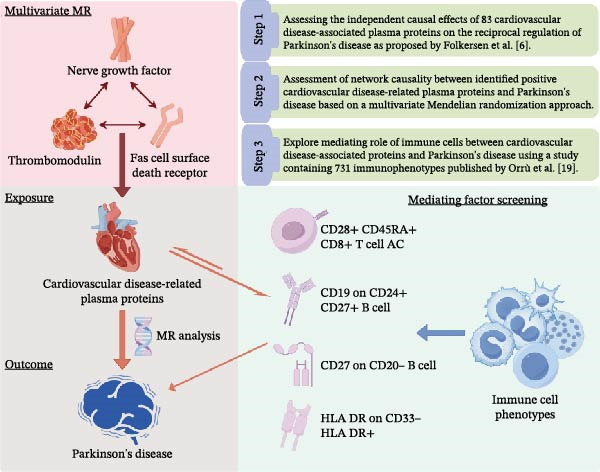
Flowchart of study design.

We utilized summary data from published genome‐wide association studies (GWAS), including 731 immune cell phenotypes and 83 CVD‐related proteins, and selected appropriate single nucleotide polymorphisms (SNPs) as instrumental variables (IVs) for MR analysis to investigate their bidirectional causal relationships (Figure 1). The application of IVs in MR analysis depends on meeting three key assumptions: (i) the selected IVs show a strong association with the exposure of interest, (ii) the IVs are not confounded by factors that affect the outcome other than exposure, and (iii) the selected IVs affect the outcome only through exposure. All data in this study are derived from previously published studies and public databases; thus, no additional ethical approval is required.

### 2.2. GWAS Data Sources

Immune cell data were derived from the GWAS study conducted by Dr. Orrù et al. [19], which involved 3757 individuals from Sardinia (Table 1). The study encompassed 731 immune phenotypes, including 118 absolute cell counts (ACs), 389 median fluorescence intensities (MFIs) reflecting the levels of surface antigens, 32 morphological parameters (MPs), and 192 relative cell counts (RCs). The MFI, AC, and RC features included B cells, CD clusters (CDCs), mature T cells, monocytes, myelocytes, TBNK (T cells, B cells, and natural killer cells), and Treg cells, while the MP features included CDC and TBNK cells. Comprehensive details of the study procedures can be found in the published research. Summary statistics for each immune trait GWAS are publicly available from the GWAS Catalog (registration numbers GCST90001391 to GCST90002121).

**Table 1:** Information on the GWAS data cohort used to conduct the MR analysis.

Data source	Population	Phenotype	Sample size	No. of cases	No. of controls
FinnGen R10 (2024)	European (Finnish)	Parkinson’s disease	412,181	4681	407,500
Folkersen et al. [6]	European (Swede et al.)	83 plasma protein biomarkers in cardiovascular disease	2639	N.A.	N.A.
Orrù et al. [19]	European (Sardinian)	731 immunophenotypes (AC/MFI/MP/RC)	3394	N.A.	N.A.

Abbreviations: ACs, absolute cell counts; MFI, median fluorescence intensity; MPs, morphological parameters; RCs, relative cell counts.

Genetic data related to PD were obtained from the FinnGen Consortium (https://r10.finngen.fi/, accessed on September 10, 2024). The study cohort comprises individuals of European descent who provided informed consent. The FinnGen research project integrates genetic data related to disease endpoints from the Finnish Biobank and Finnish National Registers (Table 1). Case identification is based on the International Classification of Diseases, Tenth Revision (ICD‐10) codes. For detailed information on participant characteristics, genotyping, imputation, and quality control, please visit the FinnGen website (https://finngen.gitbook).

CVD‐related plasma protein data come from a study conducted by Folkersen et al. [6] in 2017. The study reported the results of a GWAS of 83 proteins considered to be related to CVD, involving a total of 3394 European subjects who had at least three established CVD risk factors (Table 1). Comprehensive details of the study procedures can be found in the published research.

### 2.3. Instrumental Variable Selection

To ensure the robustness and reliability of the MR analysis, we applied the following criteria for IV selection. For SNP to be associated with the exposure, we set the IV significance level to 1 × 10^−5^. Additionally, we eliminated linkage disequilibrium (LD) among SNPs, as strong LD can introduce bias (*r* ^2^ < 0.001 and clumping distance = 10,000 kb). In cases of LD genetic variants, we selected the variant with the lowest *p*‐value associated with the exposure. Subsequently, we filtered out weak instrument variables (*F* > 10) to ensure a strong association between the IVs and the exposure. Finally, we harmonized the SNPs for the exposure and outcome to ensure consistency in the effect estimates for the same effect allele and excluded palindrome SNPs or SNPs with incompatible alleles with intermediate effect allele frequencies.

### 2.4. Statistical Analysis

We employed the inverse variance weighted (IVW) method to assess the correlation between exposure and outcome. The IVW method provides accurate and stable estimates when all IVs meet the three important assumptions. We presented our results in the form of beta values (*β*) with their standard errors (SEs) or odds ratios (ORs) with their 95% confidence intervals (CIs). Heterogeneity was measured using Cochrane’s *Q* test. Potential horizontal pleiotropy was assessed through the intercept of MR‐Egger regression and the MR pleiotropy residual sum and outlier (MR‐PRESSO). To ensure the robustness of the results, we conducted sensitivity analyses using a leave‐one‐out approach to identify any potentially influential SNPs. Furthermore, for outcomes with *p* < 0.10 in previous MR analyses, we used other MR analysis models to validate the significance of the conclusions, including the MR‐Egger method, weighted median method, and simple median method. The MR‐Egger regression adjusts for potential horizontal pleiotropy at the expense of estimation precision, while the weighted median method provides accurate estimates under the assumption that at least 50% of the IVs are valid. For CVD‐related plasma proteins and PD suggested to have a causal relationship by MR analysis, we further conducted reverse MR analysis to further infer the bidirectional causal relationship.

For loci meeting the *α* = 1e–5 significance level in the GWAS data of exposure or outcome, we extracted the gene seat information at that locus as the target gene and used the KOBAS tool for GO and KEGG enrichment analyses of the target genes (http://bioinfo.org/kobas/), visualizing the most significant pathways to explore related biological processes, cellular components, molecular functions, and signaling pathways. In addition, we imported these target genes into the String platform (https://www.string-db.org) for protein–protein interaction analysis and to construct a PPI network.

In the tests for heterogeneity and horizontal pleiotropy, *p* < 0.05 is considered statistically significant. All statistical analyses were conducted using R version 4.3.2.

## 3. Results

### 3.1. The Bidirectional Causal Effect of CVD‐Related Plasma Proteins and PD

**Figure 2:**
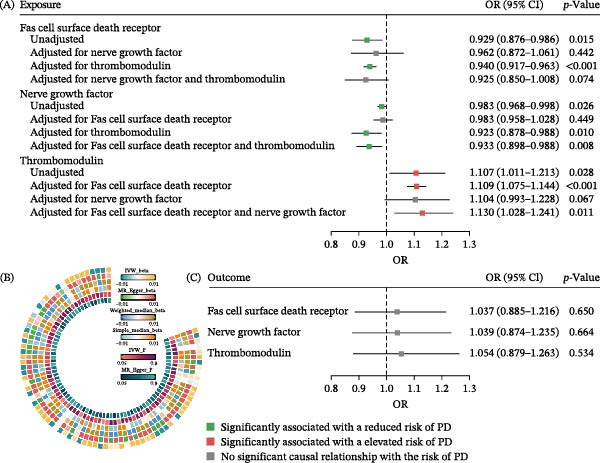
Results of Mendelian randomization with cardiovascular disease‐related proteins as exposure. (A) Forest plot of forward MR and multivariate MR results. (B) Circular heat map of sensitivity analysis using multiple MR models. (C) Forest plot of reverse Mendelian randomization.

Through a two‐sample MR analysis, we identified three CVD‐related plasma proteins associated with PD (Supporting Information: Table S1). The IVW model results showed that the Fas cell surface death receptor (OR = 0.929, 95%CI: 0.876−0.986, and *p* = 0.015) was negatively associated with an increased risk of PD. After adjusting for thrombomodulin, the causal relationship between the Fas cell surface death receptor and PD remained (OR = 0.940, 95%CI: 0.917−0.963, and *p* < 0.001). However, after adjusting for nerve growth factor (OR = 0.962, 95%CI: 0.872−1.061, and *p* = 0.442) or both (OR = 0.925, 95%CI: 0.850−1.008, and *p* = 0.074), the protective effect trend remained, but the causal association was no longer significant. Additionally, nerve growth factor (OR = 0.983, 95%CI: 0.968−0.998, and *p* = 0.026) was negatively associated with an increased risk of PD. After adjusting for thrombomodulin (OR = 0.923, 95%CI: 0.878−0.988, and *p* = 0.010) or the Fas cell surface death receptor and thrombomodulin (OR = 0.933, 95%CI: 0.898−0.988, and *p* = 0.008), the causal relationship between nerve growth factor and PD remained, but it was no longer significant after adjusting for the Fas cell surface death receptor (OR = 0.983, 95%CI: 0.958−1.028, and *p* = 0.449). Finally, thrombomodulin was positively associated with the risk of PD (OR = 1.107, 95%CI: 1.011−1.203, and *p* = 0.028). After adjusting for the Fas cell surface death receptor (OR = 1.109, 95%CI: 1.075−1.144, and *p* < 0.001) or the Fas cell surface death receptor and nerve growth factor (OR = 1.130, 95%CI: 1.028−1.241, and *p* = 0.011), the causal relationship between thrombomodulin and PD remained, but it was no longer significant after adjusting for nerve growth factor (OR = 1.104, 95%CI: 0.993−1.228, and *p* = 0.067) (Figure 2A).

In all analysis processes, there was no significant heterogeneity and horizontal pleiotropy among the IVs (Table 2). Even when switching to other MR models for sensitivity analysis, the statistical effects did not change significantly in terms of significance and direction, indicating robust analysis results (Figure 2B). In further reverse MR analysis, no reverse causal effects of PD occurrence on these three CVD‐related proteins were found (Figure 2C).

**Table 2:** Heterogeneity and horizontal pleiotropy of positive results of MR analysis with cardiovascular disease‐related plasma proteins as exposure and Parkinson’s disease as outcome.

Exposure	Heterogeneity			Horizontal pleiotropy		
	*Q*	*Q* df	*p*‐Value	Egger intercept	SE	*p*‐Value
Fas cell surface death receptor	7.79	9	0.556	−0.023	0.014	0.158
Nerve growth factor	42.62	39	0.318	0.025	0.012	0.054
Thrombomodulin	5.40	5	0.369	0.000	0.034	0.994

Abbreviation: SE, standard error.

### 3.2. Network Effects Between CVD‐Related Plasma Proteins and PD

From the positive CVD‐related plasma proteins and the exposure or outcome GWAS data with a threshold of *α* = 1e–5, we extracted information on the gene region where the SNP locus is located (Supporting Information: Table S2). Through PPI network analysis and KEGG pathway enrichment analysis, we assessed the network causal relationship between the identified positive CVD‐related plasma proteins and PD. We found that the three CVD‐related positive plasma proteins have a complex interactive network relationship with PD‐related genes, among which MAPT has the highest association with other genes (Figure 3A). It was also demonstrated that the pathways of these three plasma proteins are related to multiple sclerosis, depression, Notch pathway, and retinol metabolism, which are closely related to the occurrence of PD (Figure 3B).

Figure 3Results of biological analyses carried out with PD‐related genes and cardiovascular disease–related protein‐related genes as targets. (A) Network diagram of protein–protein interaction analysis. (B) Bar graph of KEGG pathway enrichment analysis.

**Figure:**
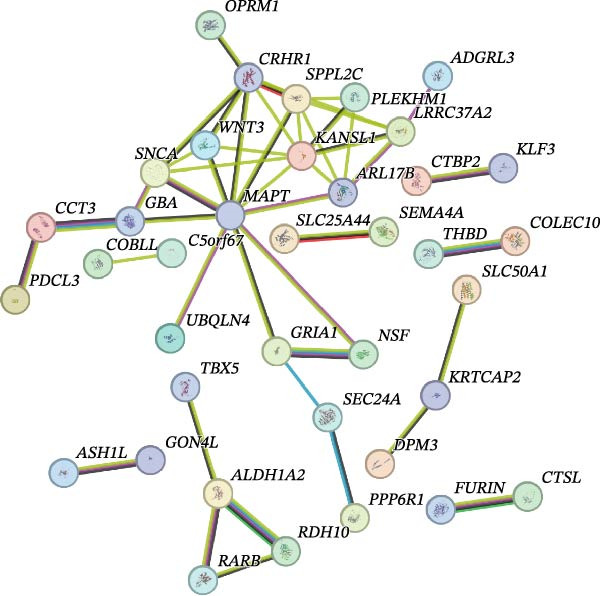
(A)

**Figure:**
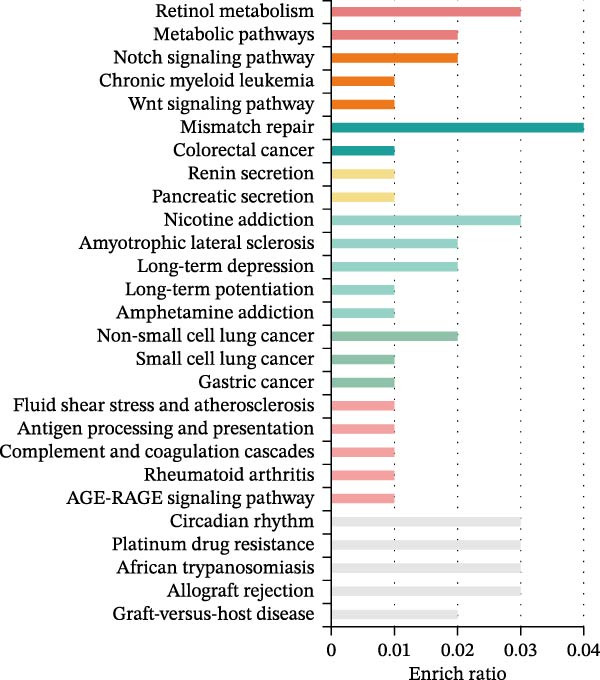
(B)

### 3.3. The Mediating Effect of Immunophenotype on the Relationship Between CVD‐Related Plasma Proteins and PD Pathogenesis

**Figure 4:**
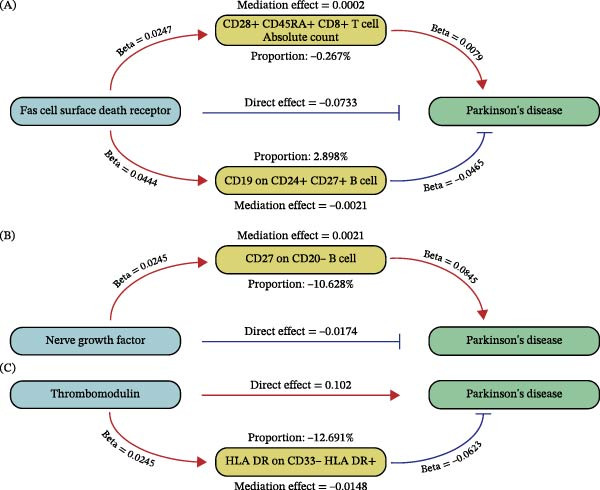
Flowchart of the potential mediation process. (A) Possible mediating pathways using Fas cell surface death receptor as an exposure. (B) Possible mediating pathways using nerve growth factor as an exposure. (C) Possible mediating pathways using thrombomodulin as an exposure.

We explored the univariate causal relationships between 731 different immune phenotypes and PD (Supporting Information: Table S3). Through mediation analysis (Supporting Information: Tables S4–S6), we identified four immune phenotypes, including CD28+CD45RA+CD8+T cell AC, that play a significant mediating role in the association between CVD‐related plasma proteins and the pathogenesis of PD. Specifically, CD28+ CD45RA+ CD8+ T cell AC had a competitive mediating effect (OR = 1.001 and 95%CI: 0.989−1.013), while CD19 on CD24+ CD27+ B cell had a partial mediating effect (OR = 0.955 and 95%CI: 0.913−0.998), accounting for −0.267% and 2.898% of the association between the Fas cell surface death receptor and PD, respectively (mediation effect = 0.0002 and −0.0021) (Figure 4A). For the association between nerve growth factor and PD, CD27 on CD20‐ B cell had a competitive mediating effect (OR = 1.088 and 95%CI: 1.037−1.142), accounting for −10.628% of the association (mediation effect = 0.0021) (Figure 4B). Additionally, HLA DR on CD33‐ HLA DR+ mediated the association between thrombomodulin and PD through a competitive mediating effect, accounting for −12.691% of the association (mediation effect = −0.0148) (Figure 4C).

## 4. Discussion

Based on a large amount of publicly available GWAS data, we explored the causal relationship between CVD‐related plasma proteins and PD at the genetic level while scrutinizing the mediating impact of 731 immune cell phenotypes. To our knowledge, this is the first MR analysis to explore the causal relationship between CVD‐related plasma proteins and PD, alongside an exploration of the mediating influence of immune cell phenotypes. Our study results identified two CVD‐related plasma proteins that have a negative causal relationship with PD and determined the significant mediating role of four immune cell phenotypes.

The Fas receptor, also known as CD95 or APO‐1, is a member of the tumor necrosis factor receptor superfamily, primarily involved in mediating apoptosis and playing a key role in the regulation of the immune system [20]. Our MR analysis results suggest a negative correlation between the Fas cell surface death receptor and an escalated risk of PD. Studies have shown that the involvement of the Fas receptor can protect dopaminergic neurons from neurotoxin‐induced damage, and treatment with Fas ligand (FasL) can significantly reduce the toxicity induced by 1‐methyl‐4‐phenyl‐1,2,3,6‐tetrahydropyridine (MPTP) in cultured mesencephalic neurons, indicating that Fas signaling has a protective effect independent of its apoptotic function [21]. Additionally, research has indicated that neuroinflammation is an important molecular mechanism in the pathogenesis of PD [22]. Microglia, the main immune cells in the central nervous system (CNS), express Fas, allowing them to respond to FasL signaling [23]. Activation of Fas has been linked to the suppression of microglial activation and a decrease in the secretion of proinflammatory cytokines [24], suggesting that Fas signaling may help to mitigate excessive inflammation in the CNS. Given its dual role in mediating apoptosis and neuroprotection, targeting the Fas signaling pathway could provide therapeutic opportunities for neurodegenerative diseases characterized by inflammation and neuronal loss. For example, small peptide antagonists that inhibit Fas activation have shown promise in providing neuroprotection in glaucoma and other disease models [25, 26].

Nerve growth factor has long played a critical role in both developmental and adult neurobiology, exerting crucial regulatory functions essential for the survival, growth, and differentiation of neural cells across the peripheral and CNSs [27]. Considered a promising therapeutic candidate for conditions like AD and PD, nerve growth factor exhibits regenerative properties specifically targeting cholinergic neurons in the basal forebrain and striatum [28]. Furthermore, nerve growth factor can stimulate mitochondrial activity and biogenesis by activating peroxisome proliferator‐activated receptor γ coactivator 1‐α (PGC‐1α) [29], a potential mechanism that is particularly important in neurodegenerative diseases where mitochondrial dysfunction is a key pathological feature [30]. Our study confirms the protective effect of nerve growth factor on PD, and continued research on the role of nerve growth factor in PD is expected to develop new therapeutic strategies aimed at slowing disease progression and improving patient outcomes.

Disruption of the blood‐brain barrier (BBB) is a hallmark of many neurodegenerative diseases, including PD and AD [31]. Notably, a prior investigation on biomarkers indicative of BBB function in AD spotlighted the heightened levels of soluble thrombomodulin (sTM) antigen and activity in AD patients, denoting BBB impairment [32]. TM may play a negative role in the pathogenesis of PD by contributing to the destruction of the integrity of the BBB. These potential mechanisms align with our MR‐derived finding indicating a positive correlation between thrombomodulin and the risk of PD. Such insights underscore the plausible impact of thrombomodulin on PD susceptibility, potentially through its involvement in BBB integrity disruption.

Through KEGG pathway enrichment analysis of genes related to three CVD‐related plasma proteins, it is possible to identify pathways that are associated with these plasma proteins and are related to amyotrophic lateral sclerosis (ALS), as well as depression, the Notch pathway, and vitamin A metabolism. ALS is also a common neurodegenerative disease [33], the Notch pathway present in the hippocampus can affect the manifestation and progression of depression [34], and vitamin A may exert a protective effect on neurons and be beneficial for neurodegenerative diseases such as PD [35]. These findings hint at a close interconnection between CVD‐related plasma proteins and neuropsychiatric ailments, warranting in‐depth exploration of the underlying mechanisms. Furthermore, the identification of antigen processing and presentation content within these pathways, linked to immunity, prompts a deeper inquiry into the potential mediating effects of immune cells. This intricate interplay underscores the need for further investigation into the intricate relationships between cardiovascular health, neuropsychiatric conditions, immune responses, and neurodegenerative diseases.

T cells and B cells are important lymphocytes in the body’s immune system. Our mediation analysis results indicate that CD28+CD45RA+ CD8+ T cell AC has a competitive mediating effect in the relationship between the Fas cell surface death receptor and PD, while CD19 on CD24+ CD27+ B cell has a partial mediating effect. CD28 provides an important second signal required for T cell activation, in addition to the primary signal from the T cell receptor (TCR), which is crucial for the full activation of T cells and promotes the proliferation and differentiation of naive T cells into effector cells [36]. T cells play a significant role in PD. Infiltration of CD8+ T cells in the CNS of patients with PD is associated with neuroinflammatory processes that can lead to neuronal damage and may exacerbate neurodegeneration [37, 38]. Similarly, T cells, as key mediators in CVDs, secrete proinflammatory cytokines that promote vascular pathology [39]. This underscores the multifaceted roles of T cells in both neurodegenerative disorders like PD and cardiovascular health, elucidating their impact on immune responses and disease progression in diverse physiological contexts.

CD19 is a B cell–specific transmembrane glycoprotein that, with its extensive cytoplasmic domain, establishes the endogenous B cell signaling threshold and may play a key role in enhancing signal transduction in multiple receptor signaling pathways [40]. Regulatory B cells (Bregs) that produce anti‐inflammatory cytokines such as IL‐10 appear to have a protective role in PD, and a higher proportion of these cells is associated with better motor scores in PD patients, suggesting that they may help to reduce inflammation and dopaminergic cell loss [41]. Yanamandra et al. [42] found that patients with PD have higher levels of antibodies against monomeric α‐synuclein in serum than controls. This suggests a potential protective role of B cell–related humoral immunity in maintaining homeostasis and clearing protein species. B cells are increasingly recognized as important players in the pathogenesis of PD. Their altered populations, potential autoimmune responses to α‐synuclein, and involvement in neuroinflammatory processes highlight their dual role as contributors to pathology and potential protectors against neurodegeneration. Further research is needed to clarify the exact mechanisms by which B cells influence the progression of PD and to explore their potential as therapeutic targets.

Additionally, we found that CD27 on CD20‐B cell has a competitive mediating effect in the relationship between nerve growth factor and PD. CD27, a costimulatory molecule, enhances B cell activation when bound to its ligand CD70, facilitating the survival, proliferation, and differentiation of activated B cells [43]. The involvement of B cells in neuroinflammation is significant, and previous studies have found that the proportion of B lymphocytes producing proinflammatory cytokines is increased in patients with PD, which may contribute to an inflammatory environment characteristic of PD pathology [44].

HLA‐DR is a major histocompatibility complex (MHC) class II molecule that plays a key role in the immune system by presenting antigens to CD4+ T cells [45]. The expression of HLA‐DR on CD33‐negative cells implies a potential involvement of these cells in antigen presentation and T cell activation, necessitating further exploration to delineate their specific functions. In CVDs, the activation of the immune system has a negative impact on patient prognosis [46], further supporting the potential mediating role of immunity between CVD‐related plasma proteins and PD.

To our knowledge, this is the first study that combines multiple bioinformatics methods to analyze the mediating role of immune cells in the association between CVD‐related plasma proteins and PD. Our study is based on published large cohort GWAS studies, including a large sample of ~200,000 individuals, which provides a high statistical power. The robustness of the IVW estimates in our study is supported by multiple MR sensitivity analyses. Stringent criteria have been implemented in our mediation analysis to mitigate potential reverse causal relationships between CVD‐related plasma proteins and to ensure the integrity and validity of the model we have constructed to elucidate the mediating effects.

However, it is necessary to acknowledge the undeniable limitations of our study. Firstly, the large GWAS studies on immune cells are currently only conducted in European populations, thereby imposing racial constraints on our research conclusions. Given the genetic diversity across various racial groups, the mediating role of immune cells ascertained in our study may manifest differently in non‐European populations. Secondly, we need to point out that MR analysis is only based on genetic level observations and causal relationship inferences and cannot replace clinical trials within the objective field. Meanwhile, due to the limitations of the MR study method, the role of time in the process of PD is ignored, and potential confounding factors may not be fully resolved. Lastly, while our study provides strong evidence for potential causal relationships at the population level, it does not elucidate the underlying biological mechanisms and lacks validation from functional studies. Future research utilizing in vitro and in vivo models is essential to verify these interactions and uncover their precise molecular pathways. Our findings can serve as key hypotheses for subsequent investigations.

Our study has important clinical application values. First, our study can provide new biomarker references for the early diagnosis and prevention of PD, such as the elevation of certain markers like TM level signaling the onset and progression of PD. Furthermore, our research underscores the intricate link between PD and CVDs, emphasizing the need for heightened attention to the potential development of cardiovascular conditions in the management and care of individuals with PD. By recognizing and addressing this interplay, there is an opportunity to enhance patient outcomes and prognosis through a comprehensive approach that considers both neurological and cardiovascular health. Moreover, the immune cell mediation analysis conducted in our study unveils promising targets and strategies for immune‐based therapies in PD, offering fresh insights into the pathogenesis and genetic underpinnings of the disease. These findings pave the way for innovative approaches to immune modulation in PD treatment and open avenues for further exploration of immune cell phenotypes in mediating other risk factors for PD across diverse racial populations. Moving forward, future researchers are encouraged to validate and extend our findings by investigating the mediating roles of immune cell phenotypes in relation to various risk factors for PD within broader and more diverse racial demographics.

## 5. Conclusion

In summary, we determined the complex causal relationship between PD and CVD‐related plasma proteins at the genetic level through comprehensive MR analysis. We identified three significant correlations between CVD‐related plasma proteins and PD and further conducted mediation analysis on 731 immune phenotypes, identifying four important mediating roles of immune phenotypes and emphasizing the complex pattern of interactions between CVD‐related plasma proteins and PD. Our research provides new biomarkers and therapeutic targets for the clinical practice of preventing and treating PD and also offers new avenues for researchers to explore the correlation between PD and CVD.

## Author Contributions

Conceptualization and design: Cai Li. Methodology: Ruotong Yao, Siyao Chen, and Yangguang Lu. Validation: Ruotong Yao, Siyao Chen, and Yangguang Lu. Formal analysis: Ruotong Yao, Siyao Chen, Yangguang Lu, Yiquan Li, and Bohuai Yu. Investigation: Cai Li, Ruotong Yao, and Yangguang Lu. Resources: Cai Li. Data curation: Ruotong Yao, Siyao Chen, Yangguang Lu, Yiquan Li, Bohuai Yu, Yingying Liu, Tingxuan Zhang, Yusheng Zhu, Feng Chen, Yuhan Lin, and Yukai Wang. Writing – original draft preparation: Ruotong Yao, Siyao Chen, and Yangguang Lu. Writing – review and editing: Cai Li, Ruotong Yao, Siyao Chen, Yangguang Lu, Yiquan Li, Bohuai Yu, Yingying Liu, Tingxuan Zhang, Yusheng Zhu, Feng Chen, Yuhan Lin, and Yukai Wang. Visualization: Ruotong Yao, Siyao Chen, and Yangguang Lu. Supervision: Cai Li. Project administration: Cai Li.

## Funding

This study was supported by the National Innovation and Entrepreneurship Training Program for College Students (Grant 202510343038) and the Student Research Project Funding Program of Wenzhou Medical University (Grant wyx2023101112).

## Disclosure

All authors read and approved the final manuscript.

## Ethics Statement

The authors have nothing to report.

## Consent

The authors have nothing to report.

## Conflicts of Interest

The authors declare no conflicts of interest.

## Supporting Information

Additional supporting information can be found online in the Supporting Information section.

## Supporting information


**Supporting Information** Table S1: Results of a Mendelian randomization analysis with 83 cardiovascular disease‐related plasma proteins as exposure and Parkinson’s disease as outcome. Table S2: Information on the gene region where the SNP locus is located extracted from positive cardiovascular disease‐associated plasma proteins and outcome Parkinson’s disease GWAS data at a threshold of *α* = 1e–5. Table S3: Results of a Mendelian randomization analysis with 731 immune cell phenotypes as exposure and Parkinson’s disease as outcome. Table S4: Results of a Mendelian randomization analysis of immunophenotypes with the Fas cell surface death receptor as exposure and positive causality with Parkinson’s disease as the outcome. Table S5: Results of a Mendelian randomization analysis of immunophenotypes with nerve growth factor as exposure and positive causality with Parkinson’s disease as the outcome. Table S6: Results of a Mendelian randomization analysis of immunophenotypes with thrombomodulin as exposure and positive causality with Parkinson’s disease as the outcome.

## Data Availability

Publicly available datasets were analyzed in this study. The raw data for this study can be obtained from FinnGen R10 (https://r10.finngen.fi/), OpenGWAS (https://gwas.mrcieu.ac.uk/), and David databases (https://david.ncifcrf.gov/).
